# Cutaneous leukocytoclastic vasculitis mimicking IgA vasculitis in a patient with intestinal Behçet’s disease: a diagnostic challenge^☆^

**DOI:** 10.1016/j.abd.2026.501315

**Published:** 2026-03-26

**Authors:** Takehiro Nakamura, Ko-Ron Chen, Toshiyuki Yamamoto

**Affiliations:** a Department of Dermatology, Fukushima Medical University, Fukushima, Japan; b Meguro Chen Dermatology Clinic, Tokyo, Shinagawa, Japan

Dear Editor,

A 34-year-old male with a history of atopic dermatitis and recurrent oral ulcers for the past two years was admitted to our department, complaining of high fever and joint pain that had started 8-days previously, which were followed 4-days later by purpura on his lower extremities. Initial examination revealed multiple palpable purpuric maculopapular lesions on the bilateral lower legs, dorsum of the feet and hands (Fig. 1 A‒B), along with disseminated papular lesions on both elbows and forearms (Fig. 2), but neither vesicles nor pustules were observed. Blood tests showed no renal dysfunction, normal serum IgA levels, and negative MPO-ANCA/PR3-ANCA. Urinalysis revealed no hematuria or proteinuria. Skin biopsy of the purpuric papular lesion on the hand showed leukocytoclastic vasculitis with fibrinoid degeneration and neutrophilic infiltration with nuclear debris in and around the vessel walls in the whole dermis (Fig. 3 A‒B). Another biopsy taken from the elbow showed perivascular neutrophil infiltration with nuclear debris in the dermis. Neither biopsy showed evidence of panniculitis. Direct Immunofluorescence (DIF) showed no perivascular deposition of C3, IgA, IgM, and IgG. On hospital day-7, the patient developed appetite loss and tested positive for fecal occult blood. Lower gastrointestinal endoscopy revealed multiple geographic ulcers in the terminal ileum (Fig. 4). On day-8, a second skin biopsy was performed to reassess the possibility of IgA vasculitis; however, DIF showed no IgA deposition in a sample from the lower limb. During hospitalization, folliculitis-like eruptions appeared on the right hand. Ophthalmological examination revealed no abnormalities. According to the Japanese Behçet's Disease Research Committee criteria, the patient was diagnosed with intestinal Behçet's Disease (BD). Oral prednisolone and infliximab were administered for intestinal symptoms, which resulted in remission of gut and skin symptoms.

**Fig. 1:**
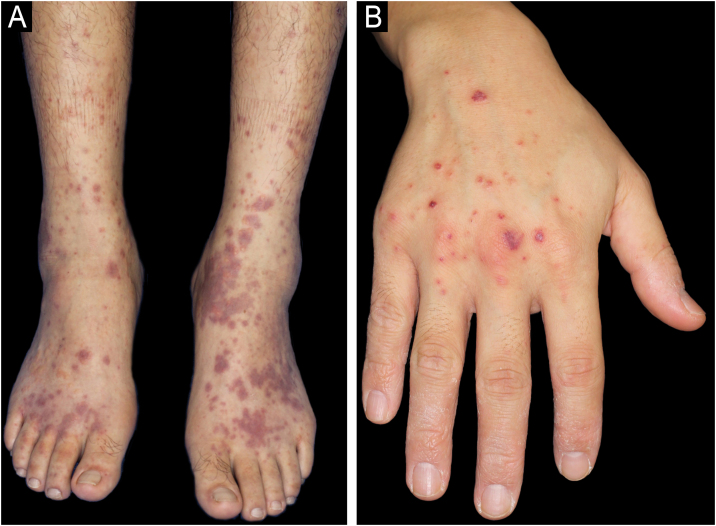
(A) Multiple purpuric maculopapular lesions extending from both the lower legs to the dorsum of the feet. (B) Hemorrhagic blisters and palpable purpuras on the dorsum of the right hand.

**Fig. 2:**
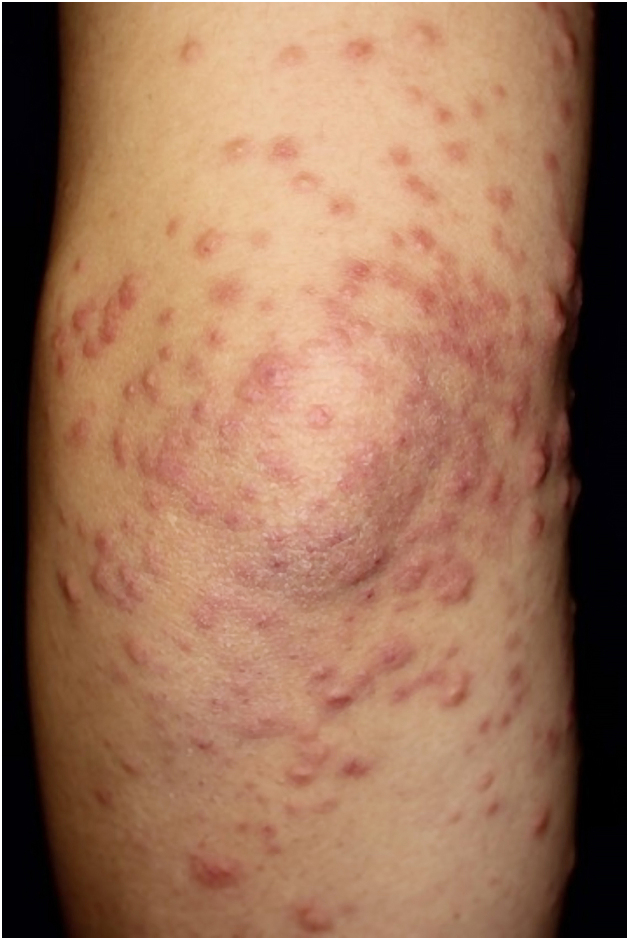
Papules in a cluster formation on the left upper extremity.

**Fig. 3:**
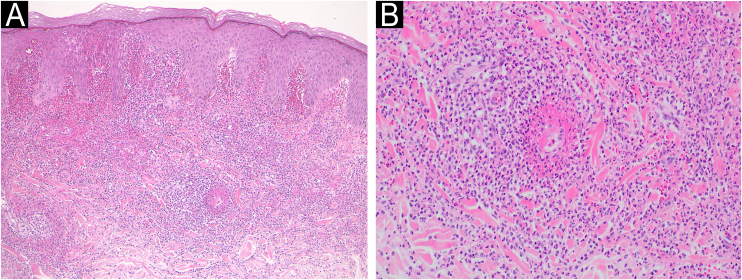
(A‒B) A biopsy taken from the lesion on the hand showing marked extravasation of red blood cells, and diffuse infiltration of inflammatory cells in the dermis and leukocytoclastic vasculitis with fibrinoid degeneration of the blood vessels in the upper to mid-dermis (Hematoxylin & eosin: A, ×100; B, ×200).

**Fig. 4:**
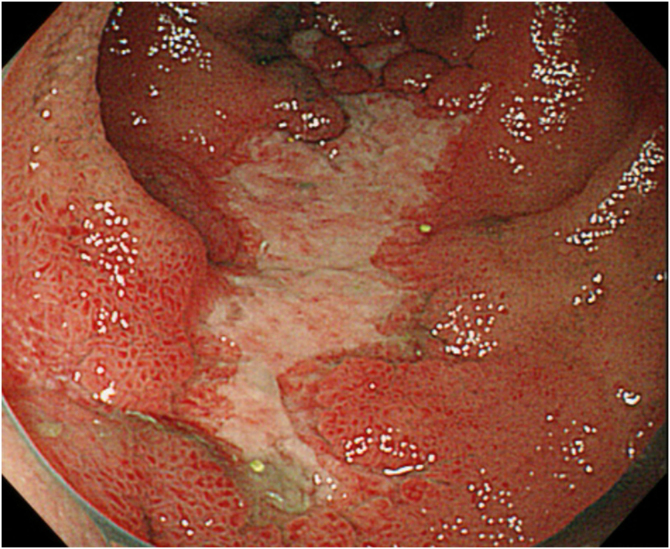
Endoscopic image: multiple geographic ulcers in the terminal ileum.

While purpura is not a typical symptom of BD,^1^ it can occasionally appear as a rare skin lesion.^2^, ^3^ A study of 20 patients with BD complicated by vasculitis reported cases in which a single patient exhibited erythema nodosum-like eruptions, palpable purpura, hemorrhagic blisters, and infiltrated erythema, suggesting that vascular inflammation occurs at various depths.^3^ On the other hand, several cases of concurrent BD and IgA vasculitis have been reported, presenting with hematuria or proteinuria, where IgA deposits were observed in both skin and kidney.^4^, ^5^ By contrast, in cases without IgA deposition, distinction between BD-associated vasculitis and IgA vasculitis is sometimes difficult. Levinsky reported high concentrations of IgA-containing immune complexes in the sera of patients with BD, suggesting that this immunological background might indicate an association between the two diseases.^6^

In the present case, IgA deposition was not observed in two times’ DIF tests at different time courses, nor were hematuria or proteinuria present. Furthermore, the multiple geographic ulcers in the terminal ileum were characteristic of BD, but not of the gut symptoms in IgA vasculitis. In conclusion, our patient developed palpable purpura caused by cutaneous vasculitis associated with intestinal BD, mimicking the clinical features of IgA vasculitis. Our case suggests atypical skin manifestations of BD, emphasizing the importance of comprehensive clinical evaluation for differential diagnosis.

## ORCID IDs

Ko-Ron Chen: 0000-0001-5429-732X

Toshiyuki Yamamoto: 0000-0002-8390-2573

## Authors’ contributions

Takehiro Nakamura: The study concept and design; data collection, or analysis and interpretation of data; writing of the manuscript or critical review of important intellectual content; data collection, analysis and interpretation; intellectual participation in the propaedeutic and/or therapeutic conduct of the studied cases; final approval of the final version of the manuscript.

Ko-Ron Chen: The study concept and design; effective participation in the research guidance; critical review of the literature; final approval of the final version of the manuscript.

Toshiyuki Yamamoto: The study concept and design; effective participation in the research guidance; critical review of the literature; final approval of the final version of the manuscript.

## Financial support

None declared.

## Research data availability

Does not apply.

## Declaration of competing interest

None declared.
